# Bird collisions with wind generators in China: a review of avoidance and minimization measures

**DOI:** 10.1007/s10661-026-15193-5

**Published:** 2026-04-13

**Authors:** Emil Friedrich

**Affiliations:** 1 Research Center for Eco-Environmental Sciences, Chinese Academy of Sciences, Beijing 100085, China; 2 Technische Universität Berlin, Berlin 10623, Germany

**Keywords:** Wind energy, Bird collisions, Mitigation measures, Chinese wind farms

## Abstract

As wind energy becomes a key pillar in global decarbonization strategies, preventing bird collisions with wind turbines has become a critical ecological challenge. This review focuses on methods for avoidance and minimization of bird collisions, with a particular emphasis on their applicability in China—the world’s largest wind energy producer. International measures to mitigate bird collision are synthesized, e.g., siting strategies, detection-reaction systems (DRSs), turbine painting, ultraviolet (UV) lighting, manual curtailment, Bluetooth-based detection, acoustic deterrents, and habitat management. Each method is evaluated in terms of technical feasibility, ecological effectiveness, and implementation challenges. While siting remains the best preventive measure, dynamic technologies such as AI-enhanced DRSs and integrated habitat management show promising potential. However, data scarcity, regulatory gaps, and limited empirical testing continue to hinder widespread adoption in China. The paper concludes by recommending multi-layered strategies that combine pre-construction planning, real-time monitoring, and post-construction habitat adaptation, alongside the need for improved national monitoring systems and context-specific field trials.

## Introduction

The global transition to renewable energy is a central pillar in the effort to reduce fossil fuel consumption and mitigate climate change (Teske, 2019). Among renewable sources, wind energy has emerged as a critical technology to national decarbonization targets. However, the ecological impacts of wind power—particularly bird collisions with wind turbines—have raised significant concerns for biodiversity conservation worldwide (Schuster et al., 2015). Mortality estimates from the USA alone suggest hundreds of thousands of bird fatalities annually (Erickson et al., 2014; Loss et al., 2013; Smallwood, 2013), while current fatality rates may be significantly higher due to the increased number of wind turbines (Choi et al., 2020). Moreover, the actual number of collisions may be underreported, as many studies are hindered by limitations in carcass detection due to factors such as limited search coverage, infrequent monitoring, and rapid carcass removal, particularly for small-bodied species (Bernardino et al., 2018; Corbeau et al., 2021; Dominguez del Valle et al., 2020; Péron et al., 2013). All these underscore the global scale of the challenge.

In response, a wide range of approaches for preventing or mitigating bird collisions have been developed. These approaches are advancing rapidly, with novel applications such as AI-enhanced detection and integrated habitat adaptation showing increasing promise. Yet, despite such progress, comprehensive evaluations of these approaches remain scarce, and most existing reviews are either dated or geographically limited (e.g., Marques et al., 2014; Corbeau et al., 2021; Weißer, 2024). A synthesis of the latest evidence and technologies is therefore needed to guide future applications.

This need to is particularly urgent in China, which is home to more than 1,500 bird species and lies at the intersection of four major global migratory bird flyways (State Council of the People’s Republic of China, 2024). Given China’s ambition to achieve carbon neutrality by 2060, comprehensive decarbonization of the energy sector will be required, making the large-scale expansion of wind energy essential (IPCC, 2023; Yang et al., 2022). This expansion, however, is also associated with increased impacts on avian species. Recent studies in China have indicated substantial avian mortality risks associated with onshore wind turbines, with estimates suggesting up to 1.36 million bird deaths annually by 2060 (Meng et al., 2024). Regional field surveys further demonstrate species-specific vulnerabilities, especially for migratory and large-bodied birds in coastal and inland flyway regions (Liu et al., 2024; Zhang et al., 2022; Zhao et al., 2024).

To address this problem, the potential for governance-based mitigation was evaluated by Liang et al. (2023), who analyzed environmental impact assessments, government documents, and field surveys across a range of provinces and landscapes. Their findings suggest that policy instruments—such as temporal restrictions on turbine operation and ecological restoration at impacted sites—may serve as effective tools to reduce the risk to soaring birds. Despite these findings, systematic post-construction monitoring remains limited, and standardized protocols for carcass detection or risk modeling are largely absent. As a result, China represents both a global hotspot for potential conflicts between wind energy expansion and biodiversity conservation, and a priority region for applying and testing up-to-date mitigation measures.

This review addresses these gaps by systematically synthesizing international approaches to bird collision avoidance and minimization, assessing their technical feasibility, ecological effectiveness, and potential for implementation in China. By integrating global experience with China-specific contexts, the review aims to provide decision-makers, developers, and researchers with an evidence-based framework to balance renewable energy development with avian biodiversity protection.

## Materials and methods

This review followed a structured literature-based approach to identify, screen, and synthesize international studies and technical sources related to bird collision mitigation at wind turbines. The methods applied for literature retrieval, selection, and supplementary data collection are described below. The review protocol was guided by established systematic review principles (Moher et al., 2009; Page et al., 2021).

### Literature search strategy

Relevant literature was identified through searches in Google Scholar, CNKI (China National Knowledge Infrastructure), and the Google search engine. Google Scholar was chosen for its wide coverage of peer-reviewed, English-language studies on bird collisions and mitigation techniques in wind energy contexts. CNKI was used to capture Chinese-language publications and to assess domestic research developments and local applicability of mitigation methods. Google Search was additionally used to identify specific commercial products related to detection-reaction systems (DRS), including company websites and technical documentation.

Search terms included combinations of: “bird collision”, “wind turbine”, and specific mitigation methods or technologies (e.g., “acoustic deterrent”, “habitat management”).

Boolean operators (AND, OR) were used to broaden or narrow the search scope as appropriate. For example: (“bird collision” OR “avian mortality”) AND (“wind turbine” OR “wind farm”) AND (“detection system” OR “deterrent method” OR “Mitigation methods”).

The main search was completed on July 1, 2025. No formal restrictions were placed on the publication date, but emphasis was given to studies published between 2000 and 2025, reflecting recent technological developments.

### Inclusion and exclusion criteria

Studies and reports were included if they:

- Focused on methods designed to prevent or reduce bird collisions with wind turbines (or closely related detection–deterrence technologies used in similar contexts such as airports)
- Provided empirical data, technical descriptions, or field applications
- Were written mainly in English, German, or Chinese

Excluded were:

- Studies unrelated to bird collision mitigation
- Duplicates or entries with insufficient information (e.g., conference abstracts without full text)

### Supplementary data collection

In addition to using Google Search to retrieve information on commercially available detection-reaction systems (e.g., IdentiFlight, DTBird) from official company websites, telephone inquiries were conducted to clarify product specifications and field deployment data.

## Results

In this chapter, several mitigation measures are presented with recent developments.

For comparison, a table of all the mitigation measures mentioned in this chapter is presented in Table 1. The readiness level describes the maturity of the evidence base and implementation stage of each mitigation method, not its ecological effectiveness. Classification was based on a qualitative review of the literature, considering (i) the general volume of published studies, (ii) whether the method has been tested at operational wind farms, and (iii) the typical stage of application reported. High readiness indicates methods commonly applied in practice and supported by multiple field studies; Medium readiness refers to approaches tested mainly in pilot projects or small-scale deployments; Low readiness denotes experimental or conceptual methods supported primarily by laboratory studies, modelling or isolated trials. Where methods are investigated at mixed stages, the category reflects the dominant status in the literature.

**Table 1:** Comparison of mitigation measures

Mitigation method	Readiness level (Implementation maturity)	Target bird species	Development in China
Siting	High	All species	Existing planning models; deployed
Habitat management	Medium	Specific species	No deployment
Painting	Medium	Specific species	No deployment
UV lighting	Low	Specific species	No deployment
Detection-reaction system	High	Specific species	Strength in AI; lack of field deployment
Manual curtailment	Medium	All species	No deployment
Bluetooth-based detection	Low	Specific species	No deployment
Acoustic deterrent	Low	Specific species	No deployment

### Siting

Proper siting of wind farms remains the best strategy to prevent avian fatalities (American Bird Conservancy, 2020; Marques et al., 2015) and is therefore a critical component of environmental impact assessments during the planning and permitting phases (Santos et al., 2018). Siting decisions can be structured into two main levels: macro-siting and micro-siting. Macro-siting focuses on identifying suitable regions based on environmental, technical, and socio-economic factors, whereas micro-siting techniques are employed by optimizing the internal turbine layout within selected areas to minimize ecological risks while considering wind direction, wake effects, and so on (Sunak et al., 2015).

GIS-based multi-criteria frameworks are widely applied in the siting process. In China, GIS-based frameworks have explicitly incorporated bird-related exclusion criteria, including major migration corridors and protected bird habitats, in order to avoid locating wind farms within areas of elevated conflict potential (Xing & Wang, 2021; Xu et al., 2020). In one case study, wintering habitats and key migration channels of whooper swans and common cranes were defined as unsuitable for wind-power development, thereby preventing wind-farm installation in large-scale avian high-use areas (Xu et al., 2020). Such macro-siting approaches are effective in improving the ecological rationality of regional wind-energy planning and in reducing structural conflicts between wind-farm development and biodiversity conservation objectives.

At the micro-siting stage, GIS is applied at spatial resolutions that allow the ecological mechanisms underlying bird–turbine collisions to be resolved. Hanssen et al. (2020) combined high-resolution digital terrain models, remote-sensing data and GPS-tracked white-tailed eagles to identify fine-scale orographic uplift hotspots. Their results show that soaring raptors preferentially use ridge systems with strong orographic uplift at flight altitudes overlapping the rotor-swept zone, which directly links these terrain features to elevated turbine collision risk.

Beyond static ecological overlays, dynamic bird monitoring systems have been proposed to complement siting assessments using radar technology, such as the MAX 3D radar system (Robin Radar Systems & Bureau Waardenburg, n.d.). At the Eemshaven site in the Netherlands, this system has been used to map real-time migration trajectories prior to construction, enabling site-specific characterization of bird movement patterns, including nocturnal migration (Kleyheeg-Hartman, 2019). Although not yet widely implemented in early-stage planning, such dynamic monitoring tools offer significant potential to enhance siting accuracy and reduce long-term ecological impacts.

### Habitat management

Habitat-based mitigation strategies aim to alter bird behavior by modifying the attractiveness of areas within or near wind farms. These approaches are broadly categorized into on-site and off-site strategies.

On-site modifications focus on reducing bird activity in the immediate vicinity of turbines. Measures include clear-cutting forest patches, removing carcasses, and eliminating food sources such as rodents or carrion near turbine bases (Marques et al., 2014; May et al., 2015). For instance, in central-eastern Spain, Pescador et al. (2019) demonstrated that tilling the soil around high-risk turbines to reduce vegetation and prey availability led to a 75–100% reduction in lesser kestrel (*Falco naumanni*) collisions.

Off-site habitat alterations promote alternative habitats outside wind farms to divert birds. Strategies include establishing feeding stations, constructing artificial nests or perches, and relocating food sources like carcass dumps (Garcia-Rosa, 2022; May et al., 2015). A suite of off-site habitat alteration strategies was implemented to mitigate collision risks for both birds in the repowering project of the Pannonia Gols and Mönchhof wind farms in Austria (Traxler, n.d.). Although such interventions have been implemented, off-site habitat intervention studies frequently report shifts in space use, but causal attribution to habitat enhancement is often confounded by concurrent construction activities, extremely small sample sizes, or the absence of pre–post collision rate comparisons (Traxler, n.d.; Walker et al., 2005; Mammen et al., 2011; Paula et al., 2011). Overall, robust evidence demonstrating that these off-site measures lead to sustained reductions in collision mortality is currently lacking, and their cost-effectiveness remains uncertain.

Both measures are highly site- and species-specific (Gómez-Catasús & Balotari-Chiebao, 2022). Integrated approaches—combining on-site risk reduction with off-site alternatives—coupled with rigorous monitoring (e.g., GPS tracking) are recommended to mitigate unintended consequences (May et al., 2015).

For China, wind power is expected to become dominant in achieving the “dual-carbon” goal (Wang et al., 2024), which also implicates that more habitats can be damaged by the establishment of wind farms (Peng et al., 2024). However, the transferability of such measures depends on species composition, land-use patterns, and the availability of long-term monitoring frameworks.

### Painting

Several recent review papers mention painting parts of wind turbines—either blades or tower bases—as a potential mitigation measure to reduce bird collisions (Asim et al., 2022; Tolvanen et al., 2023; Verma et al., 2023; Weißer, 2024). These reviews commonly refer to two experimental studies conducted in Norway that have laid the groundwork for this approach. May et al. (2020) carried out a study at the Smøla wind-power plant testing the effect of painting one rotor blade black to enhance visibility and reduce motion smear. Over more than a decade, the researchers observed a 71.9% reduction in annual fatality rates at the treated turbines compared to control turbines. In a separate study, Stokke et al. (2020) examined the impact of painting the lower 10 m of turbine tower bases black to reduce collisions with willow ptarmigan. This intervention led to a 48% reduction in recorded carcasses per search at the treated turbines.

Beyond these two Norwegian experiments, visibility-enhancing paint schemes have been implemented internationally mainly through pilot projects and small-sample field trials, with mixed and still inconclusive outcomes. In the Netherlands, a field trial led by the province of Groningen and RWE examined the effect of painting one blade black on seven turbines at Eemshaven. By early 2025, the project concluded that no significant reduction in bird strikes could be detected, a result that the authors attributed partly to the limited sample size and to insufficient visual contrast between the black blade and the heterogeneous background (RWE, 2025). However, in response to high collision rates across 30% of avian species recorded at or near the sites (Perold et al., 2020), one pilot project which tested red stripes on blades of four turbines in South Africa reported positive results (Birds & Bats Unlimited 2024, cited in Simmons et al., 2024). The color choice was approved by the Civil Aviation Authority for daytime visibility to raptors (South Africa Wind Energy Association [SAWEA], n.d.; SAWEA, BirdLife South Africa, 2023). Despite these results, there are limitations of small samples and site-specificity.

To complement current results, additional trials and large-scale studies are still ongoing, and their results will be required to determine whether turbine painting can deliver consistent and scalable reductions in collision mortality across sites and species. For example, in the UK, the Department for Environment, Food & Rural Affairs (Defra) launched a four-year program testing full-black, striped, and UV-reflective blade coatings on offshore turbines to determine which configuration most effectively reduces bird strikes (Defra, 2024; OJEU, 2024). In the Netherlands, an offshore project at Hollandse Kust West VI, led by Ecowende and partners, paints one blade red on seven turbines. The choice of red balances visibility, durability, and thermal performance (Ecowende, 2025; Vestas, 2025). In Spain, Iberdrola has implemented black blades and “eye” decals at multiple wind farms, including Cavar, Zorreras, and several sites in Burgos. Inspired partly by their use at a French airport, the decals reduced sightings of birds of prey by 65%. Iberdrola reports no impact on turbine performance and plans to expand the initiative (Iberdrola España, n.d.; Iberdrola, n.d.; Cromwell, n.d.). In the USA, a large-scale field study in Wyoming, led by Oregon State University and PacifiCorp, is assessing how black-painted blades affect collision rates for birds and bats with conclusive data unpublished yet. While data on a broader range of species are being collected, particular attention is given to behavioral responses in species such as eagles and ferruginous hawks (Oregon State University, 2024). This growing body of research, which applies the method across a wider sample of turbines and species, will be useful in assessing whether painting-based mitigation can be established as a globally effective and operationally viable solution. Such international findings can provide valuable insights into the feasibility of painting turbines for bird deterrence even though there are no documented China-specific studies or field trials on this measure.

Beyond ecological effectiveness, the choice of colors is constrained by regulatory and technical considerations. Although black is theoretically expected to provide the highest contrast on conventional white blades (Morkel et al., 2023), its application may be restricted by national regulations. In addition, thermal performance and coating durability represent practical constraints. Darker colors can increase surface heating and material stress, which has motivated the use of alternative colors such as red (Ecowende, 2025; Vestas, 2025).

In addition to color choice, pattern design introduces another layer of operational trade-offs. Both solid designs (e.g., the single-blade configuration applied at Smøla) and striped patterns (e.g., Hopefield) are considered acceptable, but may produce different outcomes under varying environmental conditions. Although no dedicated field tests of thermal load have been conducted, patterned designs are expected to reduce differential blade heating compared to solid coatings because the reflective sections limit solar absorption (Simmons et al., 2024). Consequently, wind farms located in hot climates may prefer patterned over solid designs (Simmons et al., 2024). Furthermore, alternating patterns across blades and towers have been recommended for low-light conditions to enhance detectability (Martin & Banks, 2023), indicating that pattern selection should be adapted to local climatic and illumination regimes rather than applied uniformly. Both coloring and patterning, however, increase the visibility of blades and may raise concerns about public acceptance (Morkel et al., 2023).

Painting turbine blades is operationally demanding when applied to existing turbines, requiring in situ work under calm weather conditions by specialized personnel and limiting treatment of the blade section closest to the hub. Observations from Smøla indicate that coating quality remained stable over several years, with no reported technical or social concerns (May et al., 2020). Moreover, costs are expected to be substantially lower if painting is implemented prior to construction rather than as a retrofit. To date, however, no peer-reviewed cost–benefit analyses have been published, leaving the economic feasibility of this measure largely unquantified.

### UV lightening

Many bird species possess tetrachromatic color vision, allowing them to perceive ultraviolet (UV) wavelengths beyond the human visual range. Their ocular structures transmit UV light and include photoreceptors sensitive to violet and UV spectra. This capability plays a role in key behaviors such as foraging, navigation, and signaling (Cuthill et al., 2000). These biological insights have led to growing interest in using UV light as a visual deterrent at wind turbines, based on the assumption that making turbine surfaces more visible in the UV spectrum may reduce bird collisions. In a pilot field study conducted by the Norwegian Institute for Nature Research (NINA), May et al. (2017) explored whether ultraviolet lighting could deter bird species active under low ambient light conditions from approaching wind turbines. Using UV lights with a 365 nm wavelength, the study reported a 27% reduction in bird flight activity compared to control nights. A parallel trial using violet light showed a 12% decrease. In addition, birds flew at higher altitudes under UV exposure—an average vertical displacement of 7 m—particularly for those flying below 40 m above sea level. Although these findings suggest that UV lighting may influence avian flight behavior in the vicinity of turbines, the authors caution that the study was exploratory and that further large-scale, long-term trials are needed to establish effectiveness in collision mitigation (May et al., 2017).

One of the first experimental evaluations, conducted by Young et al. (2003), tested the application of UV-reflective paint on turbine blades to reduce raptor collisions. The study reported no significant differences in avian use or mortality between painted and unpainted turbines, concluding that birds may perceive UV-reflective surfaces simply as color variations rather than as effective visual warnings. The authors also acknowledged that the low number of observed bird fatalities limited the statistical power of their findings. More recently, Cryan et al. (2021) conducted a multi-month experiment illuminating turbines with dim, flickering UV light during nighttime hours. Despite achieving full rotor coverage, the study detected no measurable changes in bird, bat, or insect activity.

According to a 2024 evaluation by Robin Radar Systems, producing a functionally strong UV effect would require higher light intensities or more precisely directed beams than those used in most trials to date (Robin Radar Systems, 2024a). However, increased brightness raises the risk of unintended consequences: could attract insects, which in turn may draw foraging birds toward turbines—potentially increasing collision risk. Robin Radar further emphasizes that the long-term effects of UV exposure on wildlife and ecosystems remain largely unknown, and that a more robust understanding of avian responses to different lighting geometries (e.g., narrow beams, flicker rates, or motion) is necessary before the approach can be considered viable (Robin Radar Systems, 2024a).

### Detection-reaction systems/automated detection systems

Advancements in sensor technology and information processing have significantly accelerated the development of detection-reaction systems (DRSs) aimed at reducing avian collisions with wind turbines. These systems operate as automated mitigation tools, using various sensors and algorithms to detect birds and trigger responsive measures in real time (Corbeau et al., 2021).

Technologies used in detection systems differ from each other. For camera-based systems, optical technologies, particularly 2D and 3D cameras, are commonly used for species detection and classification. 2D systems often rely on pixel contrast and pattern changes, whereas 3D systems such as IdentiFlight® utilize stereoscopic imagery and AI-based algorithms to assess distance, trajectory, and species identity with greater precision (Corbeau et al., 2021; Garcia-Rosa, 2022). But both can be affected by environmental factors such as fog, precipitation, and lighting conditions and thus their detection capabilities are impaired. Thermal and infrared cameras extend night-time detection, although their higher costs limit deployment (Skov, 2023). AI integration with camera feeds enables real-time or post-hoc species classification, although robust species recognition still demands extensive training datasets (Skov, 2023).

Radar systems, on the other hand, use electromagnetic waves to detect objects in motion and are particularly advantageous for long-range, wide-area monitoring. However, radar generally lacks species-specific identification and may struggle with small birds or in cluttered environments. Doppler and frequency-modulated continuous wave (FMCW) radars are among those tested for ornithological purposes (Corbeau et al., 2021; Garcia-Rosa, 2022).

To overcome the limitations of standalone technologies, some DRSs integrate radar with cameras and, in experimental setups, even microphones, as used by EchoTrack (Corbeau et al., 2021). This multi-sensor approach enhances spatial accuracy and allows species-specific reactions, such as targeted turbine curtailment (Garcia-Rosa, 2022; Skov, 2023). While promising, fully integrated commercial solutions remain limited and under development (Corbeau et al., 2021).

Despite their technical diversity, most DRSs follow a similar operational framework comprising four stages: functioning, detection, classification, and reaction. Functioning refers to the system’s temporal and spatial coverage—how continuously and thoroughly it monitors the rotor-swept zone. Detection involves the identification of a flying object using input from radar or optical systems, while classification processes the object’s attributes (e.g., speed, size) to assess collision risk. If the system determines that an object is at risk, it triggers a reaction—typically acoustic deterrents, visual signals, turbine curtailment, or combined solutions (Corbeau et al., 2021; Gradolewski et al., 2021).

The advancement of artificial intelligence (AI) has significantly enhanced the functionality of detection-reaction systems in wind farms, particularly through automated bird detection and species-specific turbine curtailment. Internationally, several AI-driven systems have been developed and deployed. For instance, MUSE AI, launched by DHI, employs video analytics and deep learning to recognize specific bird species and activate turbine slowdowns in offshore settings, thereby balancing avian safety with energy output (DHI, 2023). Similarly, ProTecBird’s AVES Wind system integrates high-resolution cameras and AI to identify protected bird species in real time, allowing responsive curtailment when birds enter a danger zone (ProTecBird, 2024). In Germany, the BirdRecorder project trained neural networks with over 16 million images, achieving over 98% identification accuracy for kites, thereby enabling reliable bird tracking and potential turbine shutdowns (Zentrum für Sonnenenergie- und Wasserstoff-Forschung Baden-Württemberg [ZSW], 2024).

While most applications remain within European wind farms, AI-based bird recognition technology has also shown promising development in China. Zhang et al. (2021) demonstrated the effective use of AI in real-time monitoring and identification of wild birds at Yuananju River National Wetland Park. Their system utilized deep convolutional neural networks for feature extraction, achieving recognition rates above 90% and real-time responsiveness below 200 ms. Although developed for wetland conservation rather than wind energy, this system illustrates the high transferability of AI-based bird detection to wind farm applications in China. These advancements underscore the critical role of AI in enhancing the accuracy, scalability, and real-time responsiveness of bird protection systems at wind farms.

With the rapid development of sensor technology and artificial intelligence algorithms, a variety of bird detection-response systems based on different technological routes have moved from experimental research to commercial applications. To provide a clearer picture of the technical characteristics of the solutions currently available in the market, Table 2 summarizes the key technical parameters and performance metrics of mainstream DRS products as of July 1, 2025.

**Table 2:** Available detection-reaction products

Product name	Company (Country)	Technologies used	Operationality	Countries using this system	Detection range	Species-level identification capability	Nighttime operation capability (Alternative product)
ProBird	Sens of Life (France)	4 K camera, sound deterrent, automatic curtailment, AI	Yes	Spain, France, Belgium, and Germany	max. 1072 m	No	No
Bird Protection System (BPS)	BIOSECO (Poland)	Stereo camera, stroboscopic light deterrent, sound deterrent, automatic curtailment, IoT, distributed computing, AI	Yes	Germany, France, Poland, and Spain	500–700 m; 360 degrees	No	No
IdentiFlight	Boulder Imaging (USA)	High-resolution stereo camera, neural network,machine vision,automatic curtailment	Yes	EU, the USA, Australia	max. 1.3 km	Yes	No
BirdVision	BirdVision GmbH & Co. KG (Germany)	Camera, AI, automatic curtailment	Yes	Germany	max. 300 m, 360 degrees	No	No
BirdSentinel	Biodiv-Wind (France)	Video-detection camera, AI, audio deterrent, automatic curtailment	Yes	France, Germany, Spain, Austria, Belgium, Finland, Iceland, etc. (12 countries)	-	No	Yes
SafeWind		Video-detection camera, AI, audio deterrent, automatic curtailment			-		
Xbird Radar		Radar, video-detection camera, AI, audio deterrent, automatic curtailment			several kilometers		
BirdRecorder	Zentrum für Sonnenenergie- und Wasserstoff-Forschung Baden-Württemberg (ZSW) (Germany)	Stereo camera, AI, automatic curtailment	No (available on the market in 2025)	-	max. 700 m	Yes (for kite)	No (Bird- und Batrecorder BBR 2.0)
RobinRadar MAX	Robin Radar System (Netherlands)	Radar (360 degrees and 3D system), high detection speed,long-term data storage, shutdown on demand module (for option)	Yes	Netherlands	3.3–10 km, 360 degrees	No	Yes
3DFlighTTrack	Diades Marine (France)	Radar (360 degrees)	Yes	-	360 degrees	No	-
BirdTrack	STRIX (Portugal)	Radar, camera, advanced algorithm, automatic curtailment	Yes	Scotland, Israel, Portugal, Spain, and Egypt	max. 12 km	Yes	-
BirdScan MV1	Swiss Birdradar (Switzerland)	Radar, adaptive magagement algorithm	Yes	France, Swizerland, Estland Germany, Kroatia, Israel, USA	max. 750 m	No	Yes
BirdScan MS1		3D scanning radar, adaptive management algorithm			max. 1.5 km, 90–360 degrees (each: 90 degrees horizontal, 40 degrees vertical)	Yes	Yes
FaunaScan MR2		Radar (frequency modulated continuous wave radar), camera, machine learning algorithm			max. 1 km for small passerines, several kms for larger birds	Yes	Yes
EchoTrack Radar-Acoustic Surveillance System	EchoTrack (Canada)	Acoustic sensing, radar, patented algorithm	yes	the USA, Canada, and South Africa	max. 4 km horizontal, max. 1.9 km vertical, 360 degrees (by radar)	Yes	Yes
Laufer Wind	Laufer Wind (the USA)	Radar, camera, automatic curtailment	No	-	max. 12 km	Yes (for eagle)	-
MUSE	DHI (Denmark)	Radar, digital camera, acoustic sensor, AI, automatic curtailment	Yes	the UK, Denmark, and the USA	max. 4 km by radar/max. 1 km by camera	Yes	No
nvbird onshore	nvisionist (Greece)	Radar, camera, AI, acoustic deterrent, automatic curtailment	Yes	Japan	max. 1 km	-	-
nvbird offshore		radar, camera, AI, acoustic deterrent, automatic curtailment			max. 10 km	Yes	-
MERLIN SCADA	DeTect (the U.S.)	Radar, acoustic monitoring, visual monitoring, automatic curtailment	Yes	The USA and other countries (under confidentiality)	max. 3–8 miles	No	Yes
SELA	ATEG Automation GmbH (Germany)	Infrared camera, automatic curtailment	Yes	Germany	max. 250 m	No	Yes
Bird Protect	Irida Ai Technologies (Greece)	Daytime camera, thermal camera, AI, acoustic deterrent, automatic curtailment	Yes	-	max. 200 m	-	Yes
SKARV	FME NorthWind (Norway)	Camera, AI, automatic curtailment	Under development	-	100–200 m	-	-
AVES Wind anti-collision system	ProTecBird + Rheinmetall Electronics GmbH	Camera, AI, automatic curtailment	-	Lithuania, Germany, Italy, Sweden, etc	-	Yes	-
DTBird	Liquen Consultoría Ambiental,S.L.(Spain)	Daylight HD camera (360 degrees), thermal camera, acoustic deterrent, automatic curtailment	Yes	Austria, Belgium, China and TW, France, Germany, Greece, Italy, Norway, Poland, Spain, Sweden, Switzerland, The Netherlands, the UK and the USA	650 m, 360 degrees	Yes	Yes
Spoor	spoor (Norway)	High-resolution camera, AI	Yes	Norway	1500 m	Yes	-

Data in Table 2 were collected from product webpages (Sens Of Life, n.d.; Bioseco, 2025, n.d.a, n.d.b; Boulder Imaging, n.d.; BirdVision, n.d.; Biodiv-Wind, n.d.a, n.d.b, n.d.c, n.d.d, n.d.e; Zentrum für Sonnenenergie- und Wasserstoff-Forschung Baden-Württemberg, 2024; Natur und Erneuerbare Energien, n.d.; RobinRadar, 2024b, 2024c; Diades Marine, 2023, n.d.; STRIX, 2025, n.d.a, n.d.b; Swiss BirdRadar Solutions AG, n.d.; EchoTrack, n.d.a, n.d.b, n.d.c; Laufer Wind Group, LLC, 2019; DHI, 2021, 2023, n.d.a, n.d.b; Nvisionist, n.d.a, n.d.b, n.d.c; DeTect, 2021, 2023, n.d.; ATEG, n.d.; Irida Technologies, n.d.a, n.d.b; FME NorthWind, n.d.; ProTecBird, 2024, n.d.; Liquen Consultoría Ambiental, S.L., 2025, n.d.; Spoor, n.d.a, n.d.b ; IdentiFlight, 2024; NorthWind Research, n.d.; Vattenfall, n.d.) and personal communications with company representatives

As a mitigation tool, DRSs offer scalable and non-intrusive alternatives to blanket curtailment strategies. Especially as the integration of AI into the systems, they can be increasingly more effective and efficient in reducing avian collisions with wind turbines. However, performance assessments remain scarce and sometimes proprietary, while several commercial DRS products claim high detection accuracy and adaptive response mechanisms. To address this issue, Ballester et al. (2024) established standardized protocols for assessing the performance of automatic detection systems used in onshore wind power plants. In the future, government agencies and wind power plant operators can refer to both these protocols and commercial DRS product information before decision-making.

### Manual observer (ornithologist) based curtailment

Manual observer-based curtailment involves trained ornithologists physically monitoring wind turbine sites to identify potential collision threats to birds in real time. Upon observing high-risk avian activity, the ornithologist communicates directly with wind turbine operators to temporarily halt turbine operations until the birds have safely moved past the area (de Lucas et al., 2012; Smallwood et al., 2009). This method relies heavily on the expertise of trained personnel who can accurately identify species and swiftly assess collision risks. Although effective in specific contexts—particularly in areas with high concentrations of endangered species or during peak migration periods—the manual approach has limitations, such as high labor costs, observer fatigue which can lead to potential human error, and restricted operational hours due to visibility constraints (May et al., 2015; Smallwood et al., 2020).

Consequently, while this method provides valuable immediate protection in targeted scenarios, it is commonly recommended as a supplementary strategy rather than a standalone long-term solution for avian collision mitigation at wind energy installations (Marques et al., 2014).

### Bluetooth-based detection systems

Bluetooth-based bird detection represents a novel and experimental approach to reducing avian collisions with wind turbines. Reddy and Namuduri (2023) developed a prototype system utilizing Bluetooth signal strength to estimate the distance between a bird-mounted transmitter and a receiver installed at a wind farm. The system triggers alerts when a tagged bird is detected at predefined distances—250 m and 175 m—from a turbine, thereby allowing operators to initiate protective measures.

However, the system also faces notable limitations. It depends entirely on prior tagging of individual birds, which restricts its applicability to only a subset of the avian population and requires substantial logistical effort. Moreover, while initial tests were promising under controlled conditions, the system’s reliability, effectiveness, and integration into turbine shut-down protocols have yet to be validated under full-scale operational environments.

### Deterrence through acoustic signals

Acoustic deterrents have increasingly been explored as methods to reduce bird fatalities at wind turbines. These approaches operate under the premise that sound—whether emitted intentionally or generated as a byproduct of turbine operation—can influence animal behavior and potentially reduce mortality risks.

The ambient noise produced by wind turbines themselves has shown behavioral effects on birds. A field experiment simulating turbine sounds on a previously undisturbed site revealed how turbine noise, which overlaps with songbirds’ auditory range, can disrupt their habitat selection and communication (Lehnardt et al., 2023). Nevertheless, the continued occurrence of collisions suggests that turbine noise alone is insufficient as a protective mechanism.

Among more targeted technologies, ultrasonic deterrents have received notable attention. These can be classified into passive and active systems. Passive approaches include aerodynamic whistles embedded into turbine blades, which generate ultrasonic frequencies during blade rotation (Sharma & Zeng, 2022); but they have so far not been investigated in experimental studies. Conversely, active ultrasonic deterrents involve the installation of devices such as powered speakers near turbine structures to emit broadband ultrasound. Current researches mostly used this approach to prevent collisions for bats but not for birds, and showed varied results across different species and studies (Arnett et al., 2013; Clerc et al., 2025; Romano et al., 2019; Zeng et al., 2025). Used to dispel birds in agriculture (Rivadeneira et al., 2018), their effects remain questionable given the varying results in different researches (Gilsdorf et al., 2002; May et al., 2015; Ogochukwu et al., 2012). Future studies might have to further test ultrasonic deterrents’ effectiveness on birds before implementation.

In contrast to ultrasonic deterrents, bioacoustic methods use natural alarm or predator calls to invoke instinctual avoidance behavior in birds. A pilot study in Nova Scotia tested predator and alarm calls and observed a slight decrease in bird presence (Dorey et al., 2019). However, the reduction was not statistically significant, and the use of predator cues raised concerns about stress (Georgiev et al., 2022). These limitations have necessitated alternative sound-based solutions that avoid such drawbacks.

One promising direction involves the use of Acoustic Startle Reflex (ASR) sounds. Georgiev et al. (2022) investigated the use of specially designed acoustic signals intended to trigger immediate avoidance responses in birds without inducing stress or physical harm. In their controlled experiments, all birds exposed to ASR stimuli exhibited behavioral responses, which were reported to be 16% stronger than responses elicited by other sound types. The authors further found that ASR signals did not elevate stress-related hormones such as cortisol, leading them to conclude that this approach may be both effective and ethically favorable for conservation purposes.

Audio signals are also increasingly integrated with visual cues. This method not only displaces animals vertically but allows site-specific targeting, offering operational flexibility and minimizing shutdowns (Werber et al., 2023). Such methods might also be applied to birds after being tested. Additionally, acoustic deterrents are incorporated by many companies providing detection-reaction systems or automated detection systems that integrate acoustic. These systems first issue auditory warnings to prevent birds from entering hazardous zones and only escalate to temporary turbine curtailment if deterrence fails (Marques et al., 2014; May et al., 2015).

Despite their potential, several challenges remain. Public acceptance is a key concern, especially for wind farms near residential areas where added noise could exacerbate community opposition (Sinclair & DeGeorge, 2016). Ecologically, some deterrents may unintentionally disturb ground-dwelling wildlife, as sounds broadcast across a wind park could disrupt avian predator-prey dynamics by driving both birds and their prey species away (Teff-Seker et al., 2022). Furthermore, given that some birds migrate across regions, different warning signals across countries may reduce their efficiency in learning different audio warnings (Khan & Haque, 2015). Khan and Haque (2015) emphasize the need for an internationally standardized framework for acoustic and visual signals. Nevertheless, achieving such consistency remains a significant challenge due to regional variations in species and regulatory approaches.

In sum, while acoustic deterrents offer promising, non-lethal tools for reducing wildlife collisions with wind turbines, their effectiveness is context-dependent and requires further field validation. Especially in underrepresented regions like China, where empirical testing is limited, pilot studies and long-term evaluations will be crucial for informed application.

## Discussion

A comprehensive evaluation of existing bird collision mitigation strategies highlights that siting remains the most crucial measure. By strategically locating wind farms away from migratory flyways and high-density bird habitats, planners can avoid collision risks before they arise. This is especially pertinent in China, which lies at the intersection of four major migratory routes and supports more than 800 migratory species. Incorporating radar-assisted migration monitoring into the siting process, as demonstrated by systems such as MAX 3D in Europe, could enhance the accuracy of pre-construction assessments. Although China already employs GIS-based multi-criteria siting frameworks (Xing & Wang, 2021; Xu et al., 2020), these models would benefit from integration with long-term radar monitoring to capture temporal variations in bird movement, particularly along the East Asian–Australasian Flyway and other bottleneck regions.

During both the construction and operational phases, wind farms increase collision risks that may cause irreversible impacts on vulnerable species (Peng et al., 2024). To minimize these risks, real-time detection systems are essential. Detection can be achieved through manual observation or by deploying camera, radar, or hybrid systems. With the rapid development of artificial intelligence (AI), detection-reaction systems can now provide species-specific identification and response, including targeted curtailment for endangered birds. However, limited transparency in proprietary model architectures, training procedures, and validation results currently constrains independent assessment of their reliability, highlighting the need for standardized reporting and third-party evaluation of AI-based detection systems. China already possesses the technical expertise to advance such systems, as evidenced by recent domestic research on automated detection technologies (Zhang et al., 2021). Until such systems become widespread, manual monitoring by trained ornithologists can serve as an interim measure, particularly in large wind farms or during peak migration periods, allowing operators to implement temporary shutdowns when high-risk events are observed.

In addition to detection, effective mitigation often requires a reaction stage—such as turbine curtailment or deterrent activation once a bird is detected approaching a rotor-swept zone. Birds differ in their sensitivity to sound and visual cues. For instance, while turbine noise may not strongly affect migratory species, it can significantly influence resident birds (Zhu et al., 2018). Species such as the red-crowned crane, which is highly sensitive to noise, tend to avoid acoustically disturbed areas (Song, 2012). This suggests that acoustic deterrents could be deployed to discourage sensitive species from approaching turbine sites. Likewise, for species with greater sensitivity to visual cues, visual deterrents such as painting turbine blades can enhance turbine visibility. Although this method is still experimental, field studies in Norway and South Africa indicate potential. Given the diversity of China’s bird populations and habitats, further field trials under local ecological conditions are recommended to determine the long-term effectiveness of these techniques.

Although detection and reaction can be implemented separately, in practice they are increasingly combined into integrated detection–reaction systems. Several commercial products, such as IdentiFlight and DTBird, already link real-time bird identification with automated responses, ranging from turbine slowdowns to the deployment of acoustic signals. With China’s advances in AI and sensor technology, similar integrated systems could be developed and localized, enabling wind farms to balance energy generation with biodiversity protection in a dynamic and adaptive way.

By contrast, other approaches reviewed in this paper currently show limited applicability. Bluetooth-based detection systems, for example, rely on tagging individual birds, which restricts their utility to small populations and involves significant logistical challenges. Similarly, ultraviolet lighting has shown inconsistent results in field trials and raises ecological concerns, such as attracting insects that may in turn increase bird presence near turbines. Habitat management also faces major limitations: while theoretically promising, it is often difficult to design and implement effectively, highly site-specific, and associated with substantial costs. Poorly planned habitat modifications can even create ecological traps, inadvertently increasing bird activity near turbines rather than reducing it. Given these constraints, such approaches are not recommended for large-scale deployment in China at present.

## Conclusions

This review demonstrates that preventing bird collisions at wind farms is a complex but solvable challenge. The evidence consistently underscores siting as the first and foremost measure, particularly when supported by radar monitoring and long-term ecological assessments. Advances in AI-powered detection and integrated detection–reaction systems represent the next frontier in mitigation, with strong potential for commercialization in China. Manual monitoring and selective deterrents, such as acoustic signals or blade painting, may serve as valuable complementary strategies, while habitat management and other experimental methods remain constrained by high costs or uncertain outcomes.

For China, the implications are twofold. Ecologically, the country’s position along four major migratory flyways and the presence of vulnerable species demand urgent adoption of evidence-based measures. Politically, alignment with the national “dual carbon” goal and the Migratory Bird Protection Action Plan offers a unique opportunity to mainstream biodiversity safeguards within renewable energy expansion. Yet, significant gaps remain: systematic carcass monitoring is limited, standardized protocols are lacking, and field trials under Chinese ecological conditions are rare.

Looking ahead, China can play a leading role by integrating radar-informed siting into wind farm planning, piloting AI-based detection–reaction systems in high-risk regions, and establishing national standards for monitoring and mitigation. International collaboration on technology validation and knowledge exchange will further accelerate progress. By embedding bird collision mitigation into both policy and practice, China has the potential not only to minimize ecological risks domestically but also to set a global example of reconciling renewable energy growth with biodiversity conservation.

## Data Availability

The datasets created and analyzed for this work are not publicly available. However, they can be obtained from the corresponding author upon justifiable request.
